# The Acute Effects of Electronic Cigarette Vaping and Tobacco Cigarette Smoking on Choroidal Thickness in Young, Healthy, Habitual, Dual Smokers

**DOI:** 10.3390/toxics8040085

**Published:** 2020-10-11

**Authors:** Olga E. Makri, Athina Pallikari, Konstantinos Kagkelaris, Stylianos N. Mastronikolis, Georgios Karanasios, Chrysanthos Symeonidis, Panagiotis Plotas, Constantine D. Georgakopoulos

**Affiliations:** 1 Department of Ophthalmology, Medical School, University of Patras, 26504 Patras, Greece; makriolga@upatras.gr (O.E.M.); pallikariathina@gmail.com (A.P.); kostas.kagkelaris@gmail.com (K.K.); mastronikst@gmail.com (S.N.M.); pplotas82@gmail.com (P.P.); 2 Department of Pharmacy, Medical School, University of Patras, 26504 Patras, Greece; up1019494@upnet.gr; 3 2nd Department of Ophthalmology, “Papageorgiou” General Hospital, School of Medicine, Aristotle University of Thessaloniki, 54124 Thessaloniki, Greece; chrys2209@gmail.com

**Keywords:** choroidal thickness, central macular thickness, dual smokers, electronic cigarettes, electronic cigarette vaping, nicotine, smoking, tobacco cigarettes

## Abstract

The present study aims to evaluate and compare the acute effects of tobacco cigarettes (TC) smoking and electronic cigarette (EC) vaping on foveal and choroidal thickness (CT) in young, healthy, dual smokers. Participants underwent four trials: 5 min TC; 5 min EC; 30 min EC; and 60 min nothing (sham trial). Scans before and immediately after each trial were obtained using spectral domain optical coherence tomography with the enhanced depth imaging mode. Changes in central foveal thickness (CFT), subfoveal choroidal thickness (SFCT), and CT at fourother points, 500 μm and 1000 μm temporally and nasally to the fovea, were measured. Forty-seven participants (33 male, 14 female; mean age 24.85 ± 1.57 years) were included. They smoked 13.53 ± 5.27 TCs/day for 6 ± 2.3 years and vaped ECs for the past 2.4 ± 1.08 years. We did not observe any statistically significant change in SFCT, CFT, and CT of the other points after any of the fourtrials. The acute changes in CFT and CT after EC vaping or TC smoking did not differ significantly compared to the sham trial. Smoking and vaping does not seem to result in statistically significant acute alterations in foveal and CT in young, dual smokers.

## 1. Introduction

Tobacco cigarette (TC) smoking has been recognized as the leading preventable cause of morbidity and premature mortality worldwide. It is associated with lung cancer, obstructive pulmonary disease, cardiovascular disease, and sudden death. Although TC smoking prevalence is declining [1], electronic cigarette (EC) vaping has aroused public health concerns due to its constantly increasing popularity, especially among youths. Actually, ECs are promoted as a safe alternative to traditional TC and are even proposed as a smoking-cessation method through the use of low nicotine or nicotine-free e-liquids. Interestingly, in their majority, EC users are concurrently TCs smokers and are characterized as dual smokers [2]. The prevalence of EC use in the United States is estimated to be 3–4% [3] while in Europe it is estimated to be 0.2–27% [2]. 

Electronic cigarette vaping seems to produce carbon monoxide and carcinogenic compounds like carbonyl species [4,5]. Furthermore, e-liquids, despite the highly variable amount of nicotine that they might contain, also contain a plethora of solvents and flavoring additives that might exhibit detrimental cellular effects, especially after exposure to the high temperatures of the vaporization process [6]. It is worth mentioning that the exact contents of e-liquids are seldom specified in detail by the manufacturers [7]. Concerns about ECs’ safety increased significantly after the outbreak of a series of potentially fatal ECs vaping-related lung injuries that were attributed to vitamin E acetate, an e-liquids additive [8]. Regarding carcinogenesis and the long-term cardiovascular and pulmonary EC-related consequences, these have not yet been completely clarified since EC remains a relatively new product [9]. It is almost certain, however, that EC vaping is not a risk-free habit [9].

In ophthalmology, TC smoking is recognized as risk factor for the development of age-related macular degeneration, diabetic retinopathy, retinal vein occlusion, anterior ischemic optic neuropathy, open angle glaucoma, cataract formation, thyroid ophthalmopathy, and several other entities [10]. Regarding EC, currently, there is no established association with possible ocular side effects.

Choroid is one of the most highly vascularized human tissues that lies between the retinal pigment epithelium and sclera [11]. It receives arterial blood from the ophthalmic artery and provides oxygen and nutrients to the fovea and the outer layers of the rest of the retina and especially to the extremely metabolically demanding photoreceptors. Furthermore, choroid exerts thermoregulatory and secretory functions, with the production of several growth factors including vascular endothelial growth factor [11]. Dysregulation of the choroidal functions has been implicated in the pathogenesis of several TC smoking-related ocular pathologies, such as age-related macular degeneration and glaucoma [11]. Consequently, the scientific interest has focused on investigating the possible effects of chronic TC smoking on the retinal or choroidal anatomy with the assumption that choroidal thickness (CT) changes depict alterations in the choroidal blood flow [12]. While there are several published studies about TC smoking that demonstrate contradictory conclusions, to our knowledge, there is no published data that specifically addresses the effects of EC vaping on CT. The purpose of our study is to evaluate and compare the acute effects of TC smoking and EC vaping on CT and central foveal thickness (CFT) in young, healthy, habitual, dual smokers.

## 2. Materials and Methods 

### 2.1. Ethics Statement

The study protocol of this prospective, cross-sectional, comparative, interventional study was approved by the Institutional Review Board of the University Hospital of Patras (No-167, 27 April 2020). The study was performed in adherence with the tenets of the Declaration of Helsinki. All the study procedures were meticulously explained and a written informed consent was obtained from all the participants.

### 2.2. Patients and Methods

Inclusion criteria were young healthy adults, aged 20–30 years, with a smoking history not exceeding 10 years who, at that time, were dual TC and EC users. Dual users were defined as smokers who used nicotine-containing ECs at least once a week for the past 3 months and smoked TCs daily the last 3 months [13]. Participants were included if they recalled normal blood pressure, heart rhythm, and normal blood parameters during their last check-up.

Exclusion criteria were best corrected visual acuity <20/20, >3 diopters spherical and >1 diopter cylindrical refractive error, history of any ocular disease or surgery, pregnancy, any systemic morbidity including cardiovascular, pulmonary, metabolic, or inflammatory diseases, and a history of taking any medication, like vasoactive drugs, within the last 3 months. Finally, candidates unwilling to provide written informed consent were excluded.

After asked to abstain from TC smoking and EC vaping, caffeine, alcohol, or food intake and to avoid any strenuous physical exercise for at least 8 h, subjects underwent 4 different trials at 4 different days as previously described [14]:

TC; smoking of one standard TC (1.0 mg nicotine, 13 mg tar) with the instruction to inhale 10 puffs during 5 min.

EC; 5 min; vaping of EC with nicotine containing e-liquid (18 mg/mL according to the package label). Participants were asked to obtain 10 puffs in 5 min.

EC; 30 min; vaping EC with nicotine containing e-liquid (18 mg/mL according to the package label) for 30 min. During thetrial, subjects were asked to obtain 10 puffs in 5 min and then vape ad lib for 25 min. 

Sham trial; no smoking or vaping for 60 min.

For standardization purposes, all participants throughout the study smoked the same brand of TC and vaped the same type of EC device (Nobacco Ephos, 900 mAH) filled with the same nicotine containing e-liquid (Nobacco BaSIS PG 18 mg, NOBACCO Company Greece) without any added flavoring or aroma, according to the package label. For hygiene reasons, disposable mouthpieces were used every time for each trial. The trials were performed in the morning between 8:00 and 10:00 to avoid CT diurnal fluctuations [15].

Scans were obtained by an experienced investigator using the Spectralis optical coherence tomography (OCT) (Heidelberg Engineering, Heidelberg, Germany, software version 5.3) with the application of the enhanced depth imaging (EDI) mode. The right eyes were examined before and immediately after each trial using the follow-up scanning protocol of the SD-OCT device where the baseline scan was used as reference image and coincided with the follow-up scan. The CFT measurements were provided automatically. Choroidal thickness measurements were performed manually by a blinded investigator who measured the perpendicular distance between the outer edge of the hyper-reflective line, corresponding to the retinal pigment epithelium, and the hyper-reflective line of the chorio-scleral interface. The CT was measured subfoveally (SFCT) and at 4 points 500 µm and 1000 µm nasally and temporally to the fovea. Three scans were acquired each time and the mean value of the 3 measurements was used for statistical analysis. 

The primary outcome variable was the SFCT change after the EC5 min, EC30 min vaping, TC smoking trials, and after the sham trial. Sham trial represents the CT fluctuations. Secondary outcome variables included the change in CFT and in CT at 500 μm and 1000 μm temporally and nasally to the fovea after each trial. We also evaluated and compared the absolute and the percentage changes that occurred after the 4 trials in SFCT, CFT, and CT of the other 4 evaluated points.

### 2.3. Statistical Analysis

Statistical analysis was performed by a blinded investigator using the SPSS 23.0 software (SPSS, Inc., Chicago, IL, USA). Normality of data was examined using the Kolmogorov–Smirnov test. The paired sample Student’s *t*-test was performed to compare parametric data and the Wilcoxon non-parametric test was used for pair-wise comparisons of non-parametric values.

For head to head comparisons, we analyzed the data from the subjects that completed all the 4 trials according to protocol. Comparisons of absolute and percentage changes were conducted with the Kruskal–Wallis test. For all statistical analyses performed, differences were considered statistically significant at *p* < 0.05.

A power analysis was conducted using the GPower software (version 3.1) and was based on the results of a previous study [16], and the minimum sample size required in this study was calculated as at least 32 participants with the significance level being set to ≤0.05 and statistical power at 0.95.

## 3. Results

The right eyes of 47 healthy dual smokers (33 male, 14 female) that met all the inclusion and none of the exclusion criteria were included in this study (Table 1). The subjects’ mean age was 24.9 ± 1.57 years (range 23–30 years). They reported smoking for 6 ± 2.3 years (range 2–10 years), approximately a mean number of 13.5 ± 5.27 TCs/day (range 3–28 TCs/day). They vaped ECs for the past 2.4 ± 1.08 years (range 1–4 years) and recalled vaping EC at least once, which determined a mean of 4.2 ± 1.38 days (range 2–7 days) during the last week.

Forty-seven participants underwent the TC and sham trial, 41 underwent the EC5min trial, and 40 participants underwent the EC30 min trial. Some participants did not complete all trials since they failed to attend the follow-up. Table 2 shows the CFT, the SFCT, and the CT at the fourother selected points at baseline and immediately after the fourtrials. Compared to baseline, we did not observe any statistically significant difference in the evaluated variables after any of the fourtrials.

Forty participants completed all of the trials according to the study protocol. The absolute and the percentage changes in SFCT, in CFT, and in the CT of the other fourevaluated points are presented in Table 3. According to our results, vaping of EC for 5 or 30 min or smoking a TC resulted in acute changes in the evaluated retinal and choroidal points that did not differ statistically significantly compared to the changes that were observed during the sham trial which, in turn, are attributed to the normal fluctuations of retinal and CT. In addition, the changes in SFCT, in CFT, and in the CT of the other selected fourpoints that occurred after the EC5 min and EC30 min vaping trials did not differ statistically significantly between them, neither when compared to the TC-induced changes. However, we have to note that at the point located 1000 μm nasally to the fovea the comparisons of the absolute and percentage changes in the CT were marginally not statistically significant. More precisely, we observed a tendency of increase in the CT at this particular point after the two EC vaping trials. Nevertheless, the percentage of those changes does not seem to be of clinical significance.

## 4. Discussion

Smoking constitutes a public health issue whereas EC use has increased substantially during the last years, since they have been promoted as a harm reduction strategy in TCs smokers [2,3]. Popularity of ECs raises concerns about their safety due to the lack of long-term studies. Several studies have tried to evaluate the effects of TC smoking on the choroidal and retinal thickness in order to enlighten the pathogenesis of TC smoking-associated ocular diseases but the results remain inconclusive. A meta-analysis on 13 observational studies (614 smokers, 625 controls) concluded that TC smoking did not cause any significant change on CFT or CT [12]. This meta-analysis however focused on the long-term effects of TC smoking.

We aimed to evaluate the acute effects of TC smoking and EC vaping on CT and CFT. We included two different EC vaping trials. The EC5min trial was selected as a direct comparison with the TC procedure, while it has been estimated that plasma nicotine levels during EC5min, with 18 mg/mL nicotine containing e-liquid, are nearly three-fold lower compared to those achieved after TC5min. The EC30min trial mimics the usual pattern of EC vaping and results in nicotine plasma levels comparable with those after TC smoking for 5 min [14,17].

Tobacco cigarette smoking is recognized as the most important modifiable risk factor for cardiovascular disease and it exerts its adverse events, at least partly, through an increase in sympathetic nerve activity [18]. In healthy smokers, smoking of the first TC of the day triggered a significant acute increase in systolic blood pressure by 7%, in diastolic blood pressure by 10%, and in the heart rate by 25% [19]. Electronic cigarette vaping also seems to produce acute cardiovascular effects. A meta-analysis compared the acute TCs and ECs cardiovascular effects and found that EC vaping resulted in an acute increase in the heart rate and systemic blood pressure, but to a lesser extent than TC smoking [18]. The authors attributed the sympathoexcitatory effects of ECs to the nicotine contained in the EC aerosol [18].

The acute effects of smoking and vaping in systemic circulation cannot necessarily apply to all organs [20]. Local metabolic and vascular effects result in inconsistent responses in several organs [21] and this particularly applies to sophisticated tissues like the retina and choroid. Retinal circulation is characterized by slow blood flow and, since retinal vessels lack autonomic innervation, is mainly dependent on local regulatory mechanisms [22,23]. More precisely, retina has local myogenic and metabolic autoregulatory mechanisms in order to maintain constant retinal blood flow regardless of ocular perfusion pressure changes and to adjust blood flow to retinal metabolic demands [22]. Conversely, choroid characterized by high blood flow does not have autoregulatory mechanisms in the strict sense of the definition. Its sympathetic innervation enables it to regulate and preserve the choroidal blood flow through an instantaneous reaction to sudden changes in perfusion pressure [22,23]. These vascular regulatory mechanisms, combined with the blood−retinal barriers, preserve a strictly controlled environment so as to enable an optimal visual function.

In situations of acute stress response, like the immediate post-smoking period, that are characterized by an acute increase in heart rate and systemic blood pressure [18,19] and acute increase in perfusion pressure, the choroidal sympathetic innervation results in immediate, compensatory vasoconstriction and increase in vascular resistance. Consequently, the mean blood flow in choroidal circulation is maintained constant, protecting the eye against over-perfusion [22,23,24]. These choroidal regulatory mechanisms are effective even in up to 67% increases in mean perfusion pressure [25].

In our study, we applied the EDI OCT in order to indirectly evaluate alterations in choroidal blood circulation by measurements of CT changes after EC vaping and TC smoking. We could not find any significant acute changes in the CT of the evaluated points immediately after TC smoking and after two different EC trials. Furthermore, the effects of the TC, EC5min, and EC30min did not differ significantly and were comparable to the sham test. Furthermore, the CFT did not change significantly after the smoking and vaping tests. Since the fovea is supplied by the choroidal circulation, a relatively stable choroidal blood flow did not affect fovea’s structure. Our findings imply that the choroidal vascular regulatory mechanisms were effective and compensated for possible acute cardiovascular effects of TC and EC in young healthy smokers.

Our results, however, do not agree with previous studies which also have yielded controversial results about the acute effects of TC smoking [26,27]. A study on healthy smokers aged 34.3 ± 8 years who smoked 13.4 ± 10.4 TCs/day for 15.4 ± 8.8 years found that TC smoking caused a significant acute CT decrease which persisted for 3 h [26]. Conversely, another study on healthy subjects with smoking histories exceeding 10 years and a consumption of 24.25 ± 4.88 TCs/day during the last year showed that smoking caused an acute, within 5 min, statistically significant increase in CT that returned to baseline levels one hour later, while no statistically significant acute change was observed in the CFT [27]. The authors attributed their contradicting results to the different OCT devices theyutilized and to the different screening and enrolment methods [27]. Another plausible explanation for these discrepancies in smoking-related CT acute changes could be the different levels of nicotine absorption among individuals and the variety of TC smoke components that are absorbed and might alter the end-organ responsiveness [20]. One, however, could not ignore that the smokers included in the above-mentioned studies might be considered chronic and even heavy smokers and the possible underling chronic smoking-induced vascular dysfunction might have resulted in abnormal choroidal vascular reactivity [28,29] and in alterations of the retinal vascular autoregulatory mechanisms [30,31].

Our study has certain strengths and limitations. Although there are no standard definitions and grading of EC use, the study was designed to include a relative homogenous sample with the enrolled participants being considered experienced EC vapers who could effectively inhale aerosol during vaping. Since the different puffing habits of TCs and ECs users may contribute to difficulty in attaining standard nicotine levels, we tried to standardize the trialsas much as possible. More precisely, we gave clear instructions for the smoking and vaping procedures and we provided the same brand and type of TC while we utilized an EC device and e-liquids of the same type and brand throughout the study in order to avoid possible confounders. Furthermore, we included two different EC vaping trials in an effort to create comparable trials with TC smoking in regard to time and nicotine absorption.

On the other hand, there were several limitations of this study that should be acknowledged. The sample size might be not adequate enough to reveal statistically significant changes. In addition, the study design excluded long-term smokers and, consequently, our results do not necessarily apply to the general smoking population with established smoking-related vasculopathy and possible comorbidities. No test with nicotine-free e-liquid was used in this study, making it impossible to assess potential effects of the other components of e-liquids. We did not measure the systemic circulation parameters like arterial pressure and heart rate changes, and we also did not measure possible changes in intraocular pressure after smoking or vaping. Consequently, the effect of ECs and TCs on ocular perfusion pressure could not be assessed. We also have to recognize that, although accepted, measurements of CT changes with the EDI SD-OCT can neither depict the ultrastructural changes, nor provide direct and objective assessment of alterations in retinal and choroidal blood circulation. Furthermore, measurement of CT was performed manually since there is no available choroidal segmentation software algorithm. We did not evaluate possible changes in thickness of more peripheral retinal points and, consequently, we could not evaluate the integrity of retinal autoregulatory mechanisms after TC or EC triggering. Finally, no serum tests were performed at baseline to confirm abstinence from nicotine or cafeine, neither after TC smoking and EC vaping in order to evaluate plasma nicotine levels and objectively compare TC and EC exposures.

## 5. Conclusions

This study aimed to evaluate the acute effects of EC vaping on choroidal circulation and to compare them with the TC smoking-induced acute effects. It seems that an intact choroidal regulatory mechanism in young dual smokers resulted in no statistically significant alterations in CMT, SFCT, and in CT in four other points. Moreover, well-designed, prospective studies enrolling more participants or even exclusive EC vapers and also evaluating the retinal autoregolatory mechanisms are required in order to assess the acute effects of EC vaping and TC smoking on choroidal and retinal circulation. Given the growing popularity and availability of newer generation EC devices with superior nicotine pharmacokinetics, we should focus particularly on the possible acute and chronic EC-related ocular effects.

## Figures and Tables

**Table 1:** Characteristics of the 47 healthy, dual smokers that were enrolled in the study.

Characteristic	Values
Sex	
Male, n (%)	33 (70%)
Female, n (%)	14 (30%)
Age (years)	24.9 ± 1.57 (range 23–30)
Years of smoking	6 ± 2.3 (range 2–10)
Number of TCs/day	13.5 ± 5.27 (range 3–28)
Years of vaping	2.4 ± 1.08 (range 1–4)
Number of days that vaped during the last week	4.2 ± 1.38 (range 2–7)

TC: tobacco cigarettes.

**Table 2:** Central foveal thickness, subfoveal choroidal thickness, and choroidal thickness at 4 points 500 μm and 1000 μm nasally and temporally to the fovea at baseline and immediately after the 4 trials.

Parameters	TC (*n* = 47)			EC5 min (*n* = 41)			EC30 min (*n* = 40)			Sham (*n* = 47)		
	**BEFORE**	**AFTER**	***p***	**BEFORE**	**AFTER**	***p***	**BEFORE**	**AFTER**	***p***	**BEFORE**	**AFTER**	***p***
**CFT**	231.7 ± 19	230.8 ± 16.7	0.416 *	229.3 ± 15.2	229.5 ± 16	0.529 *	238 ± 26.29	238.6 ± 23.9	0.182 *	228.6 ± 16.5	228.9 ± 16.4	0.237 †
**SFCT**	331.7 ± 79.1	334.9 ± 80.7	0.155 †	356.2 ± 81	357.3 ± 82.6	0.616 †	349.7 ± 80.7	354.3 ± 81.9	0.126 *	348.5 ± 92.7	347.9 ± 92.1	0.837 *
**500 μm** **temporal CT**	329.4 ± 81.3	331.3 ± 84.5	0.435 *	349.1 ± 87.5	349.7 ± 89.3	0.740 *	343.6 ± 83	344.9 ± 89	0.595 *	338.2 ± 95.9	337.3 ± 96	0.563 *
**500 μm** **nasal CT**	312.9 ± 71.8	314.5 ± 71.1	0.248 *	338.3 ± 73.6	339.7 ± 76.9	0.607 †	330.3 ± 70.1	333.9 ± 76.3	0.128 *	328.4 ± 83.5	328.5 ± 83.4	0.928 †
**1000 μm** **temporal CT**	316.2 ± 90	321.7 ± 96.9	0.438 *	346.2 ± 93.5	348.5 ± 93.3	0.350 †	339.4 ± 83.8	343.8 ± 93.1	0.096 †	338.5 ± 98.7	336.9 ± 99.1	0.357 †
**1000 μm** **nasal CT**	294 ± 65.3	295.5 ± 67.4	0.643 *	311.2 ± 68.4	316.7 ± 70	0.165 †	313.3 ± 64.4	313.2 ± 75.6	0.986 †	306.7 ± 78.8	304.2 ± 79.2	0.229 *

* Wilcoxon test, † Paired Samples *t*-test. TC: smoking of one standard tobacco cigarette for 5 min, EC5 min: vaping of electronic cigarette for 5 min, EC30 min: vaping of electronic cigarette for 30 min, Sham trial: no smoking or vaping for 60 min, CFT: central foveal thickness, SFCT: subfoveal choroidal thickness, CT: choroidal thickness.

**Table 3:** The absolute and the percentage changes in central foveal thickness, subfoveal choroidal thickness, and choroidal thickness at 4 points 500 μm and 1000 μm nasally and temporally to the fovea at baseline and immediately after the 4 trials.

	TC	EC5 min	EC30 min	SHAM	*p* *
**CFT**					
Change (μm)	−1 (−17 to +9)	+1 (−15 to +14)	+1 (−31 to +21)	1 (−3 to +4)	0.374
Percentage (%)	0 (−7 to +4)	0 (−6 to +6)	0 (−11 to +9)	0 (−1 to +2)	0.445
**SFCT**					
Change (μm)	+2 (−26 to +30)	+3 (−27 to +34)	+3 (−70 to +53)	0 (−20 to +15)	0.166
Percentage (%)	0 (−8 to +9)	+1 (−7 to +8)	+1 (−14 to +14)	0 (−6 to +5)	0.191
**500 μm temporal CT**					
Change (μm)	0 (−36 to +61)	0 (−22 to +25)	0 (−39 to +36)	0 (−21 to +16)	0.848
Percentage (%)	0 (−9 to +14)	0 (−7 to +6)	0 (−10 to +9)	0 (−7 to +6)	0.741
**500 μm nasal CT**					
Change (μm)	+3 (−62 to +44)	0 (−38 to +53)	+5 (−44 to +37)	0 (−21 to +26)	0.325
Percentage (%)	+1 (−16 to +15)	0 (−13 to +17)	+1 (−13 to +10)	0 (−7 to +9)	0.295
**1000 μm temporal CT**					
Change (μm)	0 (−24 to +88)	0 (−39 to +32)	0 (−23 to +39)	0 (−20 to +26)	0.200
Percentage (%)	0 (−6 to +19)	0 (−11 to +10)	0 (−11 to +11)	0 (−7 to +8)	0.206
**1000 μm nasal CT**					
Change (μm)	0 (−26 to +36)	+6 (−48 to +107)	+5 (−123 to +47)	0 (−31 to +14)	0.068
Percentage (%)	0 (−11 to +13)	+2 (−14 to +33)	+1 (−45 to +12)	0 (−11 to +5)	0.099

* Kruskal–Wallis.TC: smoking of one standard tobacco cigarette for 5 min, EC5 min: vaping of electronic cigarette for 5 min, EC30 min: vaping of electronic cigarette for 30 min, Sham trial: no smoking or vaping for 60 min, CFT: central foveal thickness, SFCT: subfoveal choroidal thickness, CT: choroidal thickness.
